# Wellbeing of Breastfeeding Women in Australia and New Zealand during the COVID-19 Pandemic: A Cross-Sectional Study

**DOI:** 10.3390/nu13061831

**Published:** 2021-05-27

**Authors:** Vanessa S. Sakalidis, Alethea Rea, Sharon L. Perrella, Jacki McEachran, Grace Collis, Jennifer Miraudo, Stuart A. Prosser, Lisa Y. Gibson, Desiree Silva, Donna T. Geddes

**Affiliations:** 1 Swiss Tropical and Public Health Institute, 4051 Basel, Switzerland; vanessasakalidis@gmail.com; 2 University of Basel, 4001 Basel, Switzerland; 3 Mathematics and Statistics, Murdoch University, Perth, WA 6150, Australia; alethea.rea@murdoch.edu.au; 4 School of Molecular Sciences, University of Western Australia, Perth, WA 6009, Australia; sharon.perrella@uwa.edu.au (S.L.P.); jacki.mceachran@uwa.edu.au (J.M.); stuart@oneforwomen.com.au (S.A.P.); 5 One For Women, Mt Lawley, WA 6050, Australia; gracetcollis@gmail.com (G.C.); Jenny.Miraudo@cewa.edu.au (J.M.); 6 Telethon Kids Institute, Perth, WA 6009, Australia; lisa.gibson@telethonkids.org.au (L.Y.G.); Desiree.Silva@telethonkids.org.au (D.S.); 7 The University of Western Australia, Perth, WA 6009, Australia; 8 School of Health and Medical Sciences, Edith Cowan University, Perth, WA 6027, Australia; 9 Health and Medical Sciences, The University of Western Australia, Perth, WA 6009, Australia; 10 Joondalup Health Campus, Perth, WA 6027, Australia

**Keywords:** breastfeeding, COVID-19, SARS-Cov-2, mental health, depression, anxiety, wellbeing, lactation

## Abstract

During the COVID-19 pandemic, breastfeeding women have experienced restricted access to support, placing them at increased risk of mental health concerns and limited breastfeeding assistance. This study investigated the effect of the pandemic on feeding choices and maternal wellbeing amongst breastfeeding mothers living in Australian and New Zealand. We conducted a cross-sectional online survey that examined feeding methods, maternal mental wellbeing, worries, challenges, and positive experiences during the pandemic. Most women were exclusively breastfeeding (82%). Partial breastfeeding was associated with perceived low milk supply and longer pregnancy duration during the pandemic. Reduced mental health and wellbeing was associated with lower levels of family functioning, increased perceived stress, and perinatal anxiety. Longer pregnancy duration during the pandemic was associated with lower mental health wellbeing scores, while higher perceived stress scores were reported for regions with higher COVID-19 infection rates and women with perceived low milk supply. Women reported that the pandemic resulted in less pressure and more time for family bonding, while worries about the pandemic, family health, and parenting challenges were also cited. Mental health concerns of breastfeeding women appear to be exacerbated by COVID-19, highlighting a critical need for access to mental health and broader family support during the pandemic.

## 1. Introduction

Supporting women to continue breastfeeding during the COVID-19 pandemic is a public health priority. Research to date suggests that severe acute respiratory syndrome coronavirus 2 (SARS-Cov-2) is not transmitted in breast milk [1,2,3]. Instead, there is evidence that antibodies to SARS-Cov-2 isolated in breast milk have a strong immunological response against the virus [4,5,6]. Therefore, it is globally recommended that women continue to breastfeed to improve their infants’ health and immunity during the pandemic [7,8].

Despite the recommendation to continue breastfeeding, during the COVID-19 pandemic, the mental health and wellbeing of pregnant and breastfeeding women remain a concern [9,10,11]. New mothers’ access to support has diminished, and breastfeeding support has not adapted to the new pandemic environment [12]. Mothers have taken on an increasing proportion of caring responsibilities, including parenting and broader family support roles during the pandemic. Such factors have led to mothers being disproportionally affected by the pandemic and lockdowns [13].

In Australia and New Zealand, the COVID-19 pandemic resulted in lockdowns and stay-at-home orders beginning in March 2020. Since this time, the incidence of SARS-Cov-2 cases has remained relatively low [14]. Nonetheless, long-term lockdowns and major restrictions on state and international border crossings have either remained in place or come into effect [15,16]. Such policies have led to major changes for breastfeeding mothers, including reduced in-person support from extended family and professional services, alongside increased virtual support, employment changes, and increased responsibilities at home due to business and school closures.

Psychological stress may impact the wellbeing of breastfeeding women during COVID-19. Notwithstanding the pandemic, the physiological and psychological processes experienced in the early postpartum period already place mothers at increased risk of psychological stress, anxiety, and depression. Among other maternal and infant impacts [17,18,19,20], these conditions are associated with decreases in breastfeeding self-efficacy and duration [21,22,23,24,25]. Prior to the pandemic, between 6% and 19% of Australian mothers experienced postnatal depression or anxiety [18,26,27]. International research suggests that the pandemic is associated with unprecedented increased rates of postpartum anxiety and depression [9,28,29,30,31].

Factors that are inextricably linked to maternal wellbeing, such as exercise and sleep, are also likely to have been impacted by the pandemic [29]. Moreover, fear about potential exposure, social isolation, and financial impacts are likely to reduce mental wellbeing [32]. It is unclear how these factors may be influencing breastfeeding outcomes.

This study aimed to investigate the effect of the COVID-19 pandemic on breastfeeding and maternal wellbeing in Australia and New Zealand. Specifically, we aimed to determine how daily factors have impacted mental health, stress, and anxiety and whether these have influenced breastfeeding choices and mothers’ wellbeing.

## 2. Materials and Methods

### 2.1. Participants

We conducted a longitudinal survey of breastfeeding women that was posted online via social media platforms and shared publicly between June and November 2020. Eligible participants were exclusively (receiving only breast milk) or partially (receiving breast milk and other fluids or foods) breastfeeding a healthy term infant aged 0–7 months, and living in Australia or New Zealand. Women were excluded if they could not read English, had multiple-birth infants, an infant with conditions that may affect breastfeeding, or a preterm infant (born <37 weeks gestation), if they did not report their current breastfeeding status or their infants age, if their infant was older than 7 months, or if they were living outside of Australia or New Zealand at the time of the survey. Participants provided online informed consent for the study, which was approved by the human ethics committee of The University of Western Australia (RA4206286, 23 June 2020 and RA4204023, 21 May 2020).

### 2.2. Procedure

Every four weeks for six months, participants were invited to complete an identical online questionnaire. For this cross-sectional analysis, we included data from the first survey. The questionnaire contained closed questions detailing demographic and health information of the mother and her infant, the mother’s breastfeeding history, COVID-19 behavioural questions, and open-ended questions of mother’s experiences during the pandemic. In addition, several scales were used to assess breastfeeding, maternal and infant wellbeing, family support, and financial hardship.

### 2.3. Demographic, Health Information, and Breastfeeding History

Questions were included on the mother’s demographics and health information, detailing maternal age, education, ethnicity, parity, marital status, infant age, birth details, and health conditions of both the mother and infant. Questions on the mother’s previous breastfeeding duration, nipple/breast surgery, bra size, and any self-percieved breastfeeding problems were also assessed.

### 2.4. COVID-19 Behavioural Aspects

Questions to assess the mother’s behavioural aspects associated with work, home life, and lifestyle during the COVID-19 pandemic were included. Questions detailed the mother’s current employment status and if the pandemic had changed their employment, if they were a healthcare worker, whether they were working from home, and how frequent in the last 7 days they had worked outside of home, exercised outside, or left their home. In addition, questions assessed whether partipants were avoiding friends or family over 65 years, if they were in self-isolation, and why, and how frequently they check news updates related to COVID-19.

### 2.5. Maternal and Infant Wellbeing Scales

Infant feeding practices study questionnaire (IFPS II): Breastfeeding duration, frequency, formula use, and food introduction were assessed using an adapted version of the validated IFPS II [33,34]. Mothers were asked to rate how important certain factors were in influencing their decision to stop breastfeeding on a 4-point scale.

Perceived Stress Scale (PSS): The validated [35] 10-item scale assesses how unpredictable, uncontrollable, and overloaded respondents find their lives. Participants were asked how often they felt a certain way during the past month, with 4 positively worded items and 6 negatively worded items. Higher scores indicate higher levels of perceived stress.

General functioning subscale (GF6+) of the McMaster Family Assessment Device (FAD): The 6-item subscale of the FAD tool is validated to characterise overall family functioning [36]. Scored on a 4-point scale, higher scores indicate worse family functioning [37].

Hardship scale: Financial stress was assessed using a 6-item scale previously utilised in Australia [38]. Participants answering yes to any of the questions were categorised as experiencing hardship.

The Mental Health Continuum-Short Form (MHC-SF): The MHC-SF consists of 14 items validated for assessing aspects of wellbeing. The 6-point response scale indicates the frequency of experiencing various measures of wellbeing during the past month, ranging from 1 (never) to 6 (every day). Higher scores indicate greater levels of wellbeing. MHC-SF assesses levels of social, psychological, and emotional wellbeing, and from these subscale scores, a total score is calculated categorising mental health status as flourishing, moderate, or languishing [39].

Perinatal Anxiety Screening Scale (PASS): This validated 31-item scale identifies problematic anxiety during the perinatal period. PASS consists of four subscales that measure the following: general worry and specific fears; perfectionism, control, and trauma; social anxiety; and acute anxiety and adjustment over the past month. Scored on a 4-point scale, higher scores are indicative of higher perinatal anxiety [40,41].

Brief Infant Sleep Questionnaire (BISQ): The validated tool uses 7 items to assess infant sleep patterns and parents’ perceptions of their infant’s sleep. The items include nighttime and daytime sleep duration, frequency of night waking, duration of wakefulness and sleep-onset time, settling time, and sleep concerns [42].

### 2.6. Worries and Concerns Open Text Questions

Participants completed open-text questions describing their worries, concerns, and any positive experiences resulting from the COVID-19 pandemic. The questions were adapted from the published Covid-19 BiB Cohort quantitative study protocol [43]. Participants were asked: “What are your three biggest worries right now?”; “Can you tell us about a challenge you have faced in the last two weeks?”; and “Can you tell us how lockdown has made any parts of your life easier or more enjoyable?”

### 2.7. Statistical Analysis

Generalised linear models were used to assess the factors influencing breastfeeding and maternal wellbeing during the COVID-19 pandemic. We considered five response variables: breastfeeding status (exclusive breastfeeding yes/no), PSS (high/moderate vs. low stress scores), PASS, and MHC-SF total score and categorical score (flourishing, moderate, or languishing mental health). For each response, univariate models with explanatory variables for location (region—Western Australia, Victoria, New South Wales, Rest of Australia, New Zealand), maternal factors (age, self-reported anxiety and depression, parity, number of days pregnant since 1 March 2020), infant factors (age, in childcare), breastfeeding problems (blocked ducts, sore nipples, attachment difficulties, nipple damage, mastitis, an oversupply of milk, low milk supply, nipple shield use), employment history (impacted by COVID-19, healthcare worker, employed but on maternity leave, working outside the home), feeding (introduction of complementary foods, introduction of infant formula, current intended breastfeeding duration), sleep (if infant sleep is a perceived problem, infant’s sleep duration in the day/night, and average night waking frequency), financial hardship, exercising out of the home, family functioning (GF6+ FAD), and visiting of those > 65 years of age during the lockdown. In addition, maternal wellbeing was assessed as an explanatory variable (PSS, PASS, MHC-SF total score, and categorical).

For each univariate model, variables with a *p*-value < 0.1 were retained for multivariate modelling. Missing data were accounted for with missing case analysis, and the significance level was set at 0.05. For univariate and multivariable models, model outputs (coefficient or OR, CI, and *p*-value) were reported. All quantitative data were analysed using R (R Development Core Team, 2017).

Qualitative responses were analysed thematically. Responses were coded based on theme development from the responses’ content and were further divided into sub-themes. Percentages were reported for each sub-theme concerning worry, challenges, and lockdown benefits.

## 3. Results

### 3.1. Participant Characteristics and Demographics

The cross-sectional dataset included 364 participants. Data were excluded for incomplete data on current breastfeeding status (*n* = 36), participants from other countries (*n* = 21), infants greater than 7 months old (*n* = 44), or unknown infant age (*n* = 30), leaving 233 participants. The cross-section included women who were first-time mothers (45.5%), university-educated (75.5%), and living in Western Australia (49.4%). Infants were 94 ± 57 days old (Table 1). The majority of participants were exclusive breastfeeding (82.0%), and the remaining participants were partially breastfeeding. Reported breastfeeding problems included sore nipples (33.9%), attachment difficulties (20.6%), and nipple damage (18.9%; Table 2), and reported health problems included anxiety (24.5%) and depression (10.3%). Over one-third of women worked as healthcare professionals, 78.5% were employed and on maternity leave, and their infants slept on average 9.1 ± 1.7 h at night and 5.1 ±2.3 h during the day (Table 3). 

### 3.2. Breastfeeding Outcomes

Univariate analysis showed a reduction in the odds of exclusive breastfeeding as infant age increased and in the presence of low milk supply. There was an increase in odds of exclusive breastfeeding as the duration of infant daytime sleep increased, pregnant for a longer duration during the pandemic and if the mother reported sore nipples. Multivariate analysis maintained the relationships with infant age, and low milk supply. Additionally, there were increased odds of exclusive breastfeeding among women reporting a small problem with sleep and decreased odds of exclusive breastfeeding among women who were pregnant for a longer duration during the pandemic (Table 4).

### 3.3. Perceived Stress Scale Scores

Medium and high PSS scores were grouped and compared to low scores (Table 3). High/medium perceived stress was univariately associated with 12 explanatory variables (*p* < 0.05): region (living in Victoria was associated with higher scores compared to living in Western Australia), self-reported anxiety, older infant age, a decrease when pregnant for a longer duration during the pandemic, low milk supply, poorer family functioning, increasing PASS score, increased frequency of waking, increasing time to put the baby to sleep and intended breastfeeding duration. When MHC was treated as a categorical variable, medium/high PSS was associated with being languishing or moderately mentally healthy. Additionally, lower total MHC scores were associated with increased reporting of high/medium perceived stress. Multivariate modelling maintained significant relationships with older infant age, low milk supply, total MHC, and PASS scores. Region showed that living in Victoria or New South Wales was associated with higher scores compared to living in Western Australia. Furthermore, shorter infant night sleep duration was associated with high/medium perceived stress (Table 4).

### 3.4. Mental Health Continuum-Short Form scores

MHC-SF was associated with ten explanatory variables amongst the univariate models (*p* < 0.05). Higher MHC-SF scores were observed in women who were healthcare professionals, who exercised out of the house, and were pregnant for a longer duration during the pandemic. MHC-SF score was lower for those self-reporting anxiety or depression, breastmilk oversupply, lower family functioning, older infants, reports of medium/high perceived stress, and higher perinatal anxiety scores. Five associations remained in the multivariate modelling: longer pregnancy duration during the pandemic, family functioning, medium/high perceived stress, perinatal anxiety scores, and background as a healthcare professional.

When treated as a categorical variable, univariate modelling showed that the odds of being languishing or moderately mentally healthy increased with seven explanatory variables (*p* < 0.05): self-reported anxiety, lower family functioning, financial hardship, medium/high perceived stress, and increased perinatal anxiety scores. More exercise out of the house and being on maternity leave decreased the odds. Multivariate modelling maintained significant associations with family functioning, being on maternity leave, and perinatal anxiety.

### 3.5. Perinatal Anxiety Screening Scale Score

PASS was associated with 12 of the explanatory variables in the univariate models (*p* < 0.05). Increases in maternal age, longer duration pregnant during the pandemic, exercising out of the home or background as a health professional were associated with decreased perinatal anxiety. A higher PASS score was associated with self-reported anxiety or depression, hardship, lower family functioning, increased time spent settling the infant to sleep, high/medium perceived stress, languishing/moderate MHC, and lower total MHC score. In the multivariate modelling, an increase in maternal age was associated with decreased perinatal anxiety. Self-reported anxiety and increased time taken to settle the infant to sleep, lower total MHC score, financial hardship and moderate/higher perceived stress were associated with increased perinatal anxiety.

### 3.6. Worries, Challenges, and Lockdown Benefits

Qualitative responses to open-ended questions showed women’s most cited worries were related to COVID-19 health and safety (30.2%). Participants noted worries about being unable to see family members due to lockdowns and border closures, their family’s safety and wellbeing, and their infant or children growing up without everyday social interactions (Table 5).

“I want my daughter to grow up with a strong sense of family and community but I’m not sure she will have that in our current situation.”

General family or parent health was the second most frequently cited worry (14.3%), where women commonly noted both their mental health and sleep as a concern. Financial worries (12.5%), including living off one wage as a part of everyday life and the pandemic was also cited.

Challenges most frequently experienced in the previous two weeks included parenting and relationship (24.7%) and COVID-19 challenges (15.7%). This included day-to-day parenting issues, changing relationships, and difficulties supporting family members.

“Trying to parent my toddler while juggling newborn.”

“Feeling isolated and like my daughter and myself are missing out on things like mums’ group and playgroups.”

General family/parent health worries (14.9%) were also raised. Specifically, women noted a lack of sleep, both personally and for their infant, as a recent issue.

“Negative impacts of sleep deprivation on my day-to-day life.”

Challenges with infant health (13.4%) and breastfeeding (12.9%) were also mentioned. Other less frequent worries/challenges were daily issues related to managing the household/childcare and balancing returning to work.

Two main themes emerged as benefits of the lockdown: reduced stress/pressure (35.8%) and increased family time (24.0%). Women reported less pressure to leave the house, have visitors, or deal with social interactions, and more time to slow down. The research also highlighted the increased family time due to the partner working from home (8.6%) and more partner support (9.4%), resulting in increased family bonding. Although less frequently named, health improvements resulting from being at home were noted, including women’s opportunity to exercise.

“Lockdown made our family life easier in that we didn’t have a busy calendar (e.g., social/school events) to worry about and so day-to-day life was more relaxed, and we were able to spend more time together as a family in a slower-paced environment.”

## 4. Discussion

Breastfeeding women in Australia and New Zealand were impacted both positively and negatively by the COVID-19 pandemic. Mothers continued to exclusively breastfeed during the pandemic, citing positive outcomes related to the lockdown, including less pressure, improved bonding with their infant, and more time with family. Nonetheless, mental wellbeing, perceived stress, and perinatal anxiety appeared to be impacted by day-to-day parenting and general health factors, which were exacerbated by worry and challenges resulting from the COVID-19 pandemic and lockdowns. Greater adverse effects were experienced by women that were pregnant for a longer duration during the pandemic, those living in regions with higher COVID infection rates, and mothers with perceived low milk supply. Our study highlights that quality lactation and mental health services are required to support continued breastfeeding and maternal wellbeing through future lockdowns.

Despite experiencing various problems and recent challenges associated with breastfeeding, 82% of participants were exclusively breastfeeding, suggesting the pandemic did not negatively affect breastfeeding in our study population. Instead, incidence and types of breastfeeding problems experienced were consistent with those reported prior to the pandemic [44,45,46,47,48]. Around one-fifth of participants shared commonly reported breastfeeding problems, such as sore/damaged nipples and attachment difficulties. Although only 9% experienced perceived low milk supply, which was subjectively reported by participants, it was associated with partial breastfeeding, as were older infant age and maternal perception of infant sleep as ‘not a problem’. Perceived low milk supply is known to negatively influence breastfeeding self-efficacy and duration [49,50,51]. In a recent Australian cohort [44] assessing breastfeeding problems after birth, up to 44% of women reported low milk supply. After receiving advice from a lactation consultant, women generally showed positive changes to their supply perception. A longer pregnancy duration during the pandemic was associated with partial breastfeeding. This may be related to the limited breastfeeding education and support available to women trying to prepare for and establish breastfeeding during lockdown restrictions. While infants of younger age and mothers that perceived their infants’ sleep as “a small problem” were more likely to be exclusively breastfeeding, it is possible that these factors interrelated as durations of consolidated infant sleep increase significantly between one and four months of age [52], and many dyads change from full to partial breastfeeding from around four months [53]. Despite our sample’s high rate of exclusive breastfeeding, our findings support the need for accessible professional lactation assistance during the pandemic [44], with special consideration given to women that have been pregnant for longer periods of the pandemic and those with perceived low supply.

The pandemic and lockdowns experienced in Australia and New Zealand elicited many maternal worries and challenges but also positive impacts on family life, which may have contributed to the high exclusive breastfeeding rates observed in our study. Women frequently reported that the lockdown resulted in less pressure to leave the house and deal with social interactions, providing more time to slow down, bond with their family, and gain extra support from their partner. These experiences appeared to lead to more positive family and breastfeeding outcomes. These outcomes closely match those from several European studies assessing breastfeeding during the initial lockdown [29,54]. In a cross-sectional, high socio-economic sample of Belgian mothers surveyed during the pandemic [54], 97% of mothers were still breastfeeding, and 91% of these women reported that the pandemic had not changed or impacted their feeding choices. In the UK, [29] a more differential effect was shown: 41% of women sampled felt that their breastfeeding was protected, while 27% of women struggled to get support, with some stopping breastfeeding before they were ready because of the pandemic. Women were more likely to stop breastfeeding if they gave birth during the lockdown, if they were living in difficult conditions, with lower levels of education, or of from Black and minority ethnic backgrounds. While our study included women of predominantly high socio-economic background, which may have had a protective effect on their continued breastfeeding, the studies collectively emphasise that the personal context and home situation can differently affect women’s breastfeeding choices. These impacts may be more apparent in the pandemic, and clinicians should individualise their care accordingly [54].

The importance of family support for maternal wellbeing was strongly emphasised in our quantitative findings. Lower scores of family function were associated with reduced levels of mental wellbeing. Healthy family functioning is a well-known determinant of health outcomes, with lack of partner support being the strongest risk factor for depressive symptoms amongst postpartum Australian women [26]. While our population cited increased partner support as a benefit of the lockdown, concerns were also expressed about being separated from the wider family. Data from the US [12] support the importance of the family network during the pandemic, where mothers desired all support to be provided in person, including emotional support from family and friends and professional support from healthcare providers. During the pandemic, women highlighted that all in-person support was dramatically reduced, leading to a limited social support network and increased stress levels. Support from family and health professionals, particularly in person, appears to be a crucial factor for ensuring maternal wellbeing and should continue where possible.

Our findings indicate that women experienced decrements in their mental wellbeing during the pandemic. Our sample self-reported a high prevalence of anxiety (24.5%) compared to lower rates of depression (10.3%); and more than half had at least medium levels of perceived stress. Perinatal anxiety symptoms were mild/moderate in one-third of mothers and severe in a further 9%. Furthermore, mental health functioning, perceived stress levels, and perinatal anxiety all negatively compounded each other, whereby worse mental functioning levels were associated with higher perceived stress and perinatal anxiety. While we did not directly measure anxiety and depression in our study, the self-reported levels reflect those observed prior to the-pandemic [18,26,27]. However, it was of note that the mental wellbeing of women living in Victoria and New South Wales, which were regions that experienced higher levels of COVID-19 infection, lockdowns, and social distancing measures, was more drastically affected with higher levels of perceived stress. It is of note that increased duration being pregnant during the pandemic appeared to further exacerbate negative impacts on mental wellbeing, which suggest that mothers who gave birth during the pandemic were at heightened risk of mental health issues [55]. Indeed, an accumulating body of international evidence suggests that the COVID-19 pandemic has placed new mothers at a significantly greater risk of poor mental health, highlighting the importance of mental-health screening and care during the pandemic [9,28,29,30,31].

Day-to-day challenges related to parenting appeared to influence maternal wellbeing. These challenges reflected well-known parenting issues, which may have been intensified by the lack of social support during lockdowns. Factors such as increased time taken to settle the infant to sleep and shorter infant sleep duration at night were associated with perinatal anxiety and higher stress, respectively. The qualitative data also reflected concerns with infant and maternal sleep. These findings are congruent with reports of maternal exhaustion and links to depressive symptoms in mothers of wakeful infants [52]. Although hardship and maternal employment were not shown to have a significant relationship with breastfeeding, they appeared to be important for perinatal anxiety and mental wellbeing, and in the qualitative responses, they were consistently cited as worries resulting from the pandemic and as part of day-to-day life. Our results are consistent with Australian mental health research conducted during the pandemic’s acute phases [32]. Changes to social and work function were more strongly associated with declines in mental health than exposure to the COVID-19 virus itself [32]. Interestingly, in our study, being employed/on maternity leave and background as a healthcare professional appeared protective of mothers’ wellbeing, emphasising the importance of paid family leave and financial stability to improve mothers’ mental health [12]. Our finding of lower levels of depression and anxiety amongst healthcare professionals during the COVID-19 pandemic is consistent with that of a recent European study, and it may be explained in part by health professionals’ coping strategies such as actively seeking COVID-19 information and implementing protective measures [56].

Our study was limited for several reasons. Firstly, we recruited a small sample of women who were currently breastfeeding, of which 82% were exclusive at approximately 3 months old. This rate was higher than those typically observed in the Australian breastfeeding population at 2 months (74%), 4 months (61%), and 6 months (29%), suggesting that our sample may be biased towards women who are highly motivated to breastfeed and not generalisable to all breastfeeding women [57]. Furthermore, we relied on self-reports of health conditions and breastfeeding problems, rather than real-time clinical assessments, which may have led to over-inflating the number of health and breastfeeding issues women are truly experiencing. Nonetheless, since the reporting of breastfeeding problems was similar to that observed in the general population, our sample likely reflects an accurate snapshot of the general wellbeing and feeding problems women face in the early post-partum period in Australia and New Zealand.

## 5. Conclusions

This study provides new evidence on the impact of the COVID-19 pandemic on breastfeeding and maternal wellbeing in Australia and New Zealand. Our results highlight a high rate of continued breastfeeding during the pandemic, which was potentially influenced by the consequences of reduced stress and enhanced partner support during the lockdown. Nonetheless, breastfeeding mothers also experienced mental health issues, challenges, and worries as part of their everyday life, which may have been further exacerbated by the pandemic’s impact on support and resilience. Stronger policies and actions that enable mothers to access their immediate support networks remain essential, especially during the early post-partum period, as the pandemic continues. Particular consideration should be given to those who have been pregnant for longer periods of a lockdown, regions with higher rates of COVID infection, and mothers with perceived low milk supply. Furthermore, aiding women in lockdowns with mental health services remains critical. This cross-sectional study and future longitudinal observations of breastfeeding women during the pandemic will form the basis for developing more effective care to improve breastfeeding women’s wellbeing during current and future lockdowns.

## Figures and Tables

**Table 1:** Demographics and participant characteristics.

Variable	Mean ± SD or Count (%)
*Maternal characteristics*	
Maternal age (years)	33 ± 4.1
Parity	
Primiparous	106 (45.5%)
Multiparous	127 (54.5%)
Marital status	
Married or de facto	227 (97.4%)
Never married or de facto	5 (2.1%)
Separated or divorced	1 (0.4%)
Region	
Western Australia	115 (49.4%)
Victoria	60 (25.8%)
New South Wales	22 (9.4%)
Rest of Australia	16 (6.9%)
New Zealand	20 (8.6%)
Education	
Bachelor degree or above	176 (75.5%)
Certificate level IV	13 (5.6%)
Certificate level I–III	7 (3.0%)
Diploma	19 (8.2%)
High school	18 (7.7%)
Ethnicity	
Aboriginal or Torres Strait Islander	3 (1.3%)
Australian	176 (75.1%)
Other	54 (23.6%)
*Infant Characteristics*	
Infant age (days)	94 ± 57
Birth gestation (weeks)	39 ± 1.1
Birth weight (g)	3400 ± 430.2
Birth length (cm)	50.1 ± 2.3

SD: Standard deviation.

**Table 2:** Breastfeeding and infant sleep characteristics.

Variable	Mean ± SD or Count (%), Missing
*Breastfeeding*	
Breastfeeding frequency (per 24 h)	9.0 ± 2.8, 48
Planned breastfeeding duration (months)	16 ± 8.2, 0
Timing of introduction of complementary foods (months)	5.2 ± 0.8, 209
Breastfeeding status	
Partial breastfeeding	42 (18%)
Fully breastfeeding	191 (82%)
Introduced complementary food	
No	18 (7.7%)
Yes	24 (10.3%)
Not applicable—fully breastfeeding	191 (82.0%)
Introduced infant formula	
No	23 (9.9%)
Yes	19 (8.2%)
Not applicable—fully breastfeeding	191 (82.0%)
Breastfeeding problems	
Sore nipples	79 (33.9%), 0
Nipple damage	44 (18.9%), 0
Attachment difficulties	48 (20.6%), 0
Nipple shield use	40 (17.2%), 0
Blocked ducts	26 (11.2%), 0
Mastitis	26 (11.2%), 0
Low milk supply	20 (8.6%), 0
Oversupply	31 (13.3%), 0
*Infant Sleep*	
Nighttime sleep duration (h)	9.1 ± 1.7, 22
Daytime sleep duration (h)	5.1 ± 2.3, 22
Night waking frequency	2.6 ± 1.5, 23
Time infant spent awake between feeds (min)	31.7 ± 36.2, 22
Time taken to settle infant to sleep (min)	35 ± 28.8, 22
Perception of infant’s sleep as a problem	
Not a problem at all	120 (51.5%)
A small problem	84 (36.1%)
A very serious problem	7 (3.0%)
Missing	22 (9.4%)

SD: standard deviation.

**Table 3:** Maternal health issues, wellbeing scales scores and categories, and COVID-19 behaviours.

Variable	Mean ± SD or Count (%), Missing
*Maternal Health Issues*	
Anxiety	59 (25.3%)
Depression	24 (10.3%)
Diabetes (diagnosed before this pregnancy)	5 (2.1%)
Fertility issues requiring assisted reproduction for this pregnancy	16 (6.9%)
Thyroid disorder	8 (3.4%)
Polycystic ovary syndrome	17 (7.3%)
Insulin resistance	4 (1.7%)
No health conditions	123 (52.8%)
Other	31 (13.3%)
*Wellbeing Scales*	
Hardship	
No	187 (80.3%)
Yes	48 (20.6%), 0
GF6+ FAD ^1^ score	9.7 ± 3.4, 0
Mental Health Continuum-Short Form score	
Emotional (score: /15)	12.5 ± 2.4, 0
Social (score: /25)	13.4 ± 5.2, 0
Psychological (score: /30)	22.5 ± 5.1, 0
Mental Health Continuum-Short Form categories	
Flourishing	102 (43.8%)
Languishing	4 (1.7%)
Moderately mentally healthy	127 (54.5%)
Perceived Stress Scale score	16.1 ± 6.4, 0
Low (score: 0–13)	87 (37.3%)
Medium (score: 14–26)	135 (57.9%)
High (score: 27–40)	11 (4.7%)
Perinatal Anxiety Screening Scale score	16.1 ± 6.4, 0
Minimal anxiety symptoms (score: 0–20)	124 (53.2%)
Mild‚ moderate symptoms (score: 21–41)	76 (32.6%)
Severe anxiety symptoms (score: 42–93)	21 (9.0%)
Missing	12 (5.2%)
*COVID-19 Behaviours*	
Employment impacted by COVID	
No	220 (94.4%)
Yes	13 (5.6%)
Background as a healthcare professional	
No	145 (62.2%)
Yes	88 (37.8%)
Employed and on maternity leave	
No	50 (21.5%)
Yes	183 (78.5%)
Exercise outside of home in the last 7 days	
No	37 (15.9%)
Yes	196 (84.1%)
Avoid contact with someone over 65 years	
No	139 (59.7%)
Yes	63 (27.0%)
Not applicable	31 (13.3%)
Infant in childcare	
No	228 (97.9%)
Yes	5 (2.1%)

^1^ General functioning subscale of the McMaster Family Assessment Device. SD: standard deviation F.

**Table 4:** Univariate model and multivariate models for exclusive breastfeeding, PSS score, MSH score, and category, and PASS score.

		**Univariate Modelling #**			Multivariate Modelling *		
**Response** **(Observations in Multivariate Model)**	**Variables**	**Coeff/OR**	**CI**	***P*** **-Value**	**Coeff/OR**	**CI**	***P*** **-Value**
**Exclusive breastfeeding** 211	Intercept	-	-	-	5000	230, 11000	<0.001
	Infant age (30 days)	0.93	0.91, 0.96	<0.001	0.31	0.18, 0.51	<0.001
	Breastfeeding problems (low milk supply)	0.14	0.05, 0.37	<0.001	0.07	0.03, 0.27	<0.001
	Breastfeeding problems (sore nipples)	2.5	1.1, 5.8	0.028			
	Day sleep duration	1.2	1.0, 1.5	0.019			
	Sleep a problem			0.081			0.042
	A small problem	20	17, 23	0.025	3.6	1.2, 11	0.019
	A very serious problem	4.7	0.53, 8.4	0.211	1.4	0.20, 10	0.724
	Days pregnant during pandemic (30 days)	1.4	1.1,1.7	0.007	0.47	0.27, 0.82	0.006
**PSS ^4^** 208	Intercept	-	-	-	30	0.69, 1300	0.071
	Maternal health (anxiety)	2.1	1.1, 4.1	0.030			
	Infant age (30 days)	1.2	1.0, 1.3	0.033	1.3	1.0, 1.6	0.030
	Breastfeeding problems (low milk supply)	6.0	1.3, 17.2	0.018	6.0	0.89, 36	0.042
	GF6+ FAD ^1^ Score	1.1	1.0, 1.3	0.003			
	Settle to sleep (per 30 min)	1.4	1.0, 2.1	0.037			
	Frequency night waking	1.3	1.0, 1.6	0.014			
	Night sleep duration	0.86	0.71, 1.0	0.090	0.71	0.54, 0.94	0.015
	MHC-SF ^2^	0.92	0.89, 0.95	<0.001	0.93	0.89, 1.0	0.003
	MHC-SF categories	2.4	1.4, 4.3	0.001			
	PASS ^3^	1.1	1.1, 1.2	<0.001	1.1	1.1, 1.2	<0.001
	Region			0.034			0.029
	Region (New Zealand)	1.6	0.60, 4.5	0.325	1.3	0.31, 5.1	0.741
	Region (NSW)	1.9	0.71, 5.1	0.195	3.9	1.1, 14	0.033
	Region (Rest of Australia)	1.9	0.62, 5.1	0.243	2.5	0.56, 11	0.223
	Region (Victoria)	2.9	1.4, 6.0	0.003	3.7	1.4, 9.7	0.006
	Intended breastfeedingduration	1.0	1.0, 1.1	0.042			
	Days pregnant duringpandemic (30 days)	0.85	0.72, 0.99	0.039			
**MHC-SF** 221	Intercept				61	58, 65	<0.001
	Maternal health (anxiety)	−5.6	−8.6, −0.2	<0.001			
	Maternal health (depression)	−4.7	−9.5, −0.1	0.041			
	Infant age (30 days)	−0.82	−1.5, −0.066	0.031			
	Breastfeeding problems(oversupply)	−4.4	−8.5, −0.2	0.039			
	Health care professional	4.2	1.3, 7.1	0.005	3.2	0.75, 5.6	0.010
	Exercise outside	0.69	−0.01, 1.4	0.049			
	GF6+ FAD score	−1.3	−1.7, −0.93	<0.001	−0.9	−1.3, −0.6	<0.001
	PSS^4^ category	−8.4	−11, −5.6	<0.001	−3.4	−6.1, −0.60	0.016
	PASS	−0.37	−0.46, −0.29	<0.001	−0.22	−0.31, −0.12	<0.001
	Days pregnant during pandemic (30 days)	0.83	0.01, 1.64	0.043	0.78	0.10, 1.5	0.023
**MHC-SF** **Category** 221	Intercept	-	-	-	0.27	0.09, 0.9	0.025
	Maternal health (Anxiety)	1.9	1.0, 3.6	0.040			
	Employed (maternity leave)	0.42	0.21, 0.84	0.013	0.42	0.20, 0.89	0.021
	Exercise outside	0.84	0.74, 0.96	0.011			
	GF6+ FAD score	1.2	1.1, 1.3	<0.001	1.1	1.0, 1.3	0.008
	Hardship	2.0	1.0, 4.1	0.044			
	PSS category	2.4	1.4, 4.3	0.001			
	PASS	1.1	1.0, 1.1	<0.001	1.1	1.0, 1.1	<0.001
**PASS** 208	Intercept	-	-	-	49	34, 64	<0.001
	Maternal age	−0.93	−1.4, −0.46	<0.001	−0.54	−0.90, −0.17	0.004
	Maternal health (anxiety)	14	10, 19	<0.001	9.0	5.4, 12.5	<0.001
	Maternal health (depression)	11	5.3, 18	<0.001			
	Exercise outside	−1.0	−2.0, −0.084	0.030			
	GF6+ FAD score	1.2	0.70, 1.8	<0.001			
	Hardship	9	4.2, 14	<0.001	3.8	−0.033, 7.7	0.049
	Health care professional	−5.9	−10, −1.9	0.003			
	Settle to sleep (per 30 min)	0.11	0.040, 0.18	0.018	0.065	0.014, 0.12	0.012
	PSS category	15	11, 18	<0.001	8.2	4.8, 12	<0.001
	MHC-SF	−0.69	−0.85, −0.53	<0.001	−0.41	−0.56, −0.26	<0.001
	MHC-SF category	9.2	5.4, 13	<0.001			
	Days pregnant duringpandemic (30 days)	0.0-1.2	−2.4, -0.12	0.028			

# Includes univariate variables, which were retained for multivariate analysis (*p*-value <0.1); * Includes variables which were *p-*value <0.05 in the final multivariate model. OR: odds ratio; CI: confidence interval. ^1^ General functioning subscale of the McMaster Family Assessment Device; ^2^ Mental Health Continuum—Short Form score; ^3^ Perinatal Anxiety Screening Scale score; ^4^ Perceived Stress Scale score.

**Table 5:** Worries, challenges, and lockdown benefits cited by participants.

**Theme**	**Count (%)**	**Count (%)**
	Worries	Challenges
COVID-19 health and safety	199 (30.2)	45 (15.7)
General family /parent health	94 (14.3)	43 (14.9)
Financial	82 (12.5)	11 (3.8)
Parenting and relationships	71(10.7)	71 (24.7)
Infant/child health	76 (11.6)	38 (13.4)
Returning to work	57 (87)	16 (5.5)
Day to day household/living	51 (7.6)	25 (8.7)
Breastfeeding	28 (4.3)	37 (12.9)
Total	658	287
	Lockdown benefits	
Reduced stress/pressure	91 (35.8)	
Family time	61 (24.0)	
Working from home	22 (8.6)	
Partner support	24 (9.4)	
No change/worse	19 (7.5)	
Health	14 (5.4)	
Safety	14 (5.5)	
Breastfeeding	9 (3.5)	
Total	254	

## Data Availability

The data presented in this study are available on request from the corresponding author. The data are not publicly available due to privacy reasons.
